# Reconsidering the goals of evolution education: defining *evolution* and *evolutionary* literacy

**DOI:** 10.1186/s12052-022-00180-4

**Published:** 2022-12-19

**Authors:** Kostas Kampourakis

**Affiliations:** Section of Biology and IUFE, University of Geneva, Geneva, Switzerland

**Keywords:** Evolution education, Science literacy, Scientific literacy, Evolution literacy, Evolutionary literacy

## Abstract

In this paper, I argue that for both theoretical and practical purposes, it is useful for science education to clearly distinguish between science content knowledge and skills on the one hand, and the competencies related to their application in everyday life. This can be based on a distinction made by Douglas Roberts between two visions of literacy, and it can be effectively reconceptualized as the distinction between two types of literacy relevant to science: *Science literacy*, which is literacy relevant to the processes and products of science, related to the content of science taught in classrooms (literacy about issues within science); and *Scientific literacy*, which is literacy relevant to questions that students may encounter as citizens and to the socio-ethical implications of scientific knowledge (literacy about the implications of science for society). Based on this, we can in turn distinguish between two types of literacy related to evolution: *Evolution literacy*, which is literacy relevant to the evolution content taught in classrooms; and *Evolutionary literacy*, which is literacy relevant to questions that students may encounter as citizens and to the socio-ethical implications of scientific knowledge. In this article I argue that whereas a lot of attention has been given to evolution literacy as a learning goal, there has been less reflection and discussion about evolutionary literacy—and it is exactly the distinction between these two types of literacy that helps one realize this. Teaching for evolutionary literacy requires specific skills from teachers, which go beyond their ability to teach concepts and explanations. My aim is to initiate a discussion about how to set evolutionary literacy as a learning objective at schools along evolution literacy. A key issue in such a case is how we could prepare teachers who would be capable, and confident, to address issues going beyond the typical science content, and which are often related to worldviews, in the classroom.

## Introduction

Whereas there is a general agreement among scientists and educators that teaching evolution ought to have a prominent place in school science curricula and that learning evolution should be a main goal of science education, it should not be taken for granted that such learning makes significant contributions to literacy about science. Rather, it is necessary to clearly define what learning about evolution contributes to this kind of literacy. To achieve this, teachers need to set specific learning goals as well as to be given guidelines about how to attain those. To figure these out, it is necessary to begin reconsidering what literacy with respect to science actually consists of.

In its fundamental sense, literacy was defined by UNESCO in 1958 as “the ability to read and write, with understanding, a short, simple sentence about one’s everyday life”. In 1978, the definition was revised as “A person is functionally literate who can engage in all those activities in which literacy is required for effective functioning of his group and community and also for enabling him to continue to use reading, writing and calculation for his own and the community’s development.” (Oxenham 2008). Therefore, literacy refers to a continuum of reading, writing and numeracy skills, which depends on context and which is developed through learning in schools and in other settings. This is important to keep in mind, as it has been argued that reading and writing are not just tools for the storage and transmission of scientific knowledge. “Rather, the relationship is a constitutive one, wherein reading and writing are constitutive parts of science. Constitutive relationships define necessities because the constituents are essential elements of the whole. Remove a constituent, and the whole goes with it.” (Norris and Phillips 2003, p. 226).

In 2007, Douglas Roberts made a crucial distinction between two visions of scientific/science literacy, which represent the extremes of a continuum:

- Vision I: literacy relevant to “… the products and processes of science itself … literacy within science”
- Vision II: literacy relevant to “… situations with a scientific component, situations that students are likely to encounter as citizens … literacy about science-related situations in which considerations other than science have an important place at the table.”

This view was further elaborated a few years later by Roberts and Bybee (2014) to two types of literacy relevant to science:

- Vision I literacy: “literacy, in this view, is within science—general familiarity and fluency within the discipline, based on mastering a sampling of the language, products, processes, and traditions of science itself.”
- Vision II literacy: “begins by looking outside science… what counts as scientific literacy is learning how science fits appropriately with such personal and societal perspectives for a more complete grasp of the issues”.

Roberts and Bybee considered that Vision I should be used as default because historically, it has been the dominant model for curriculum, its measures are less complex than those of Vision II, which in turn might seem as too broad to be taught in schools and require that teachers master new content, as well as teaching and discourse styles. Eventually they suggested that “…there is more need than ever to develop ways to balance science literacy (Vision I) and scientific literacy (Vision II) in science education programs that can successfully meet the needs of all students”. This is where they make explicit and clear the distinction between science literacy and scientific literacy, which is the focus of the present article. This distinction can be represented as follows:

- Science literacy: literacy relevant to processes and products of science (literacy about issues within science).
- Scientific literacy: literacy relevant to questions that students may encounter as citizens and to the socio-ethical implications of scientific knowledge (literacy about the implications of science for society).

Table 1 provides some examples of these two kinds of literacy in various science domains.

**Table 1:** Examples of science and scientific literacy in various science domains

Domain	Science literacy	Scientific literacy
Heredity	Learning about the transmission of DNA and chromosomes across generations	Understanding that not all of our genealogical ancestors are also our genetic ancestors, and so our ancestry is only partially reflected in our DNA
Molecular biology	Learning about gene expression	Understanding how complex disease is affected by genes, and thus evaluate the results of genetic tests and the related ability to predict the possible onset of a disease
Cell biology	Learning about the proliferation of cells	Understanding how the uncontrolled proliferation of cells can result in cancer
Ecology	Learning about the CO_2_ cycle in ecosystems	Understanding the extensive impact of human activities on the environmental levels of CO_2_ and the implications for climate change
Physics	Learning about the nature of X-ray radiation	Understanding the dangers and benefits from X-rays done for medical purposes
Earth science	Learning about plate tectonics	Understanding how to behave in the case of an earthquake, and what measures need to be taken in constructions to avoid the collapse of buildings
Chemistry	Learning about the nature of polymers such as plastic	Understanding how the accumulation of plastic results in pollution

Whereas Table 1 provides examples of science and scientific literacy in various domains, I argue that evolution and evolutionary theory merit a special consideration. One might argue that in every domain we can make a distinction between science literacy and scientific literacy as defined above. Therefore, one could make the distinction between science and scientific literacy in every single discipline or domain, such as genetics, cell biology, molecular biology, etc., as in Table 1. But what I suggest in the present paper is more than distinguishing between science and scientific literacy within a discipline. When it comes to evolutionary theory, this is not enough to cover its implications because these touch upon various philosophical issues, such as worldviews, which are necessary to consider during teaching. Whereas for most science disciplines we can easily distinguish between science literacy being about science itself, and scientific literacy being about the implications of the respective science for society, evolutionary theory goes well beyond that to have various additional implications for worldviews. These implications can only become evident, I suggest, if we clearly distinguish between two types of literacy relevant to evolutionary biology:

- Evolution literacy: literacy relevant to processes and products of evolutionary biology.
- Evolutionary literacy: literacy relevant to questions that students may encounter as citizens and to the philosophical implications of scientific knowledge for one’s worldviews.

**Fig. 1:**
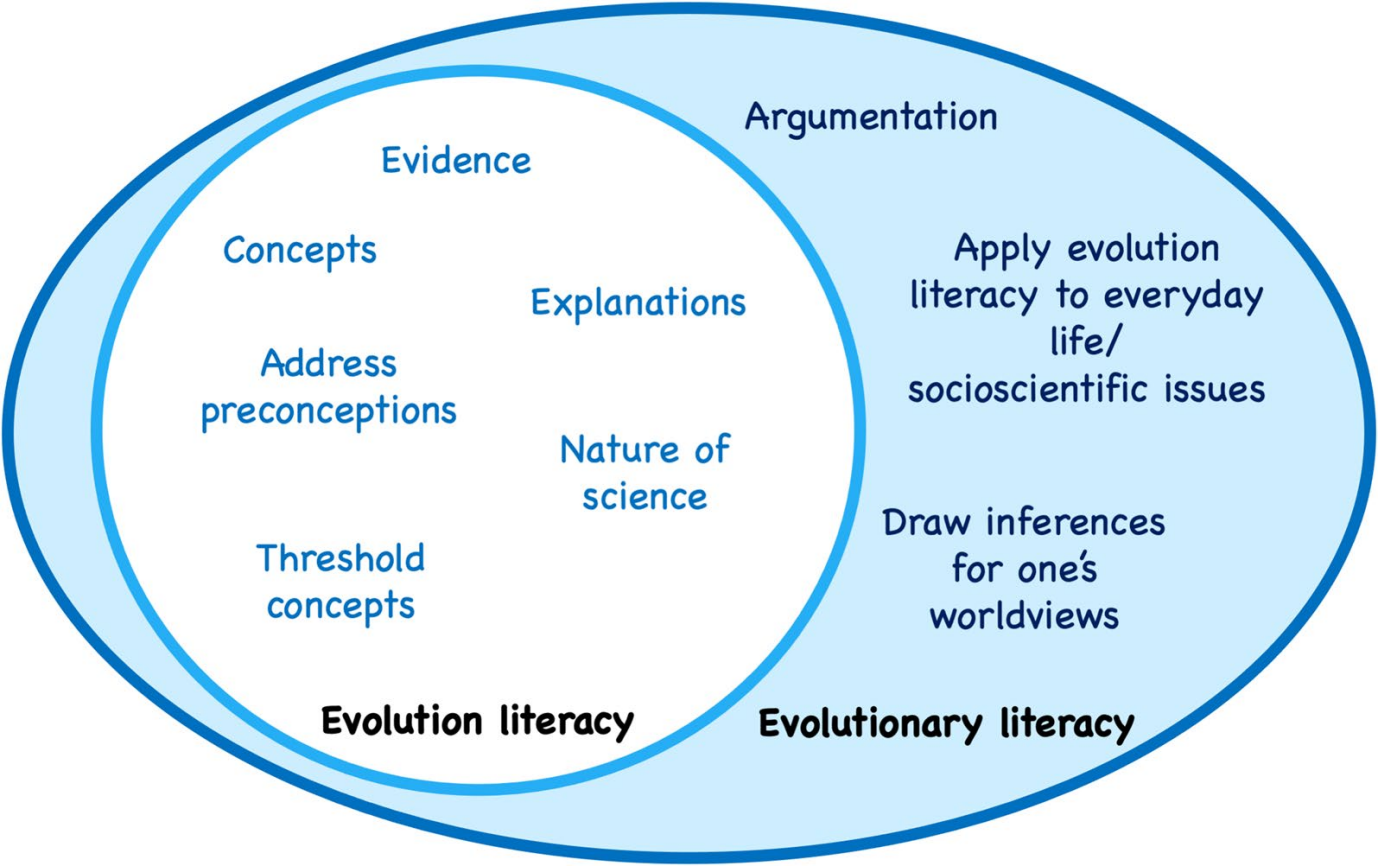
The relation between evolution and evolutionary literacy, and some of their major components

It is the last element, the philosophical implications of scientific knowledge for one’s worldviews, which we do not find in most other disciplines (and when we do it is not as strong as in evolutionary theory) that makes this distinction necessary to be made clear. When it comes to teaching, evolution is a topic that requires careful additional consideration compared to other science topics (see Reiss 2019 for such an argument). Figure 1 provides a representation of this relation. Evolution literacy is represented there as an essential, integral component of evolutionary literacy. In what follows I describe the components of evolution and evolutionary literacy in detail.

## Evolution literacy

In its most fundamental sense, evolution literacy is about learning and understanding evolutionary theory. There is so much research on the teaching and learning of evolution that is impossible to review here. Currently, there is sufficient understanding about what is missing and what ought to be done (see for instance, Glaze and Goldston 2015). A recent systematic analysis of the literature has supported the conclusion that there is a mismatch between how pedagogical content knowledge (PCK) related to evolution is framed and topics taught at the college level (Ziadie and Andrews 2018). There is work to be done, but we know where we ought to go and, often, how this could be done.

From a pedagogical perspective, evolution literacy would first require documenting and addressing students’ preconceptions about evolution. Several studies have documented these preconceptions in detail, especially about natural selection (for a review see Gregory 2009). Several studies have shown that students intuitively think of nature in terms of purpose and design, with essentialist and teleological explanations given for organisms and their features (Kampourakis 2020a). What is especially problematic is that these intuitions are based on an underlying design stance that makes them think of organisms and their parts in terms of purposes, goals, need and design (Kampourakis 2020b). These intuitions may be even enhanced by the way adaptations are presented in documentary films (Aldridge and Dingwall 2003). Therefore, to achieve evolution literacy it is necessary to address the design stance, and doing so in secondary school may be too late. There is evidence that young children do not clearly distinguish between organisms and artifacts (e.g., Kampourakis et al. 2012), and I would argue that a first step towards understanding evolution would be for young children to understand the differences between entities that are designed, such as artifacts, and entities that are not designed, such as organisms and non-living natural objects (clouds, rocks etc.). The key conclusion in this case would be that organisms are not perfectly adapted to their environments. Whether this would work, remains to be seen as there has been no empirical research on that so far.

Once this was done, the next step would be to present the evidence for evolution. The structural and functional similarities between humans and apes had been evident since the nineteenth century (e.g., Huxley 1863), and clues for our divergence from our closest relatives are evident in our chromosomes as human chromosome 2 corresponds to two distinct chromosomes in the apes (Yunis and Prakash 1982; see also Chapter 13 of Kampourakis 2018, about how this might have occurred). However, teachers also need to know that human evolution is difficult for some students to accept, something that also affects their understanding of evolution (Grunspan, et al. 2021). Furthermore, all the available evidence today from comparative anatomy, embryology, physiology, genomics, as well as from cell and molecular biology confirms all the main tenets of evolutionary theory (Coyne 2009). Then one could focus on teaching and learning some main concepts of evolutionary theory such as common ancestry, the evolutionary network of life, homology and common descent, homoplasy and convergence, evolutionary developmental biology, adaptation and natural selection, stochastic events and processes in evolution, speciation, extinction, macroevolution, and more (Kampourakis 2020a).

Finally, it is also important to consider the structure and nature of evolutionary explanations, and the related historicity of nature. The historical nature of evolutionary explanations is important for students to understand, as it differs from other kinds of scientific explanations. Historical explanations are about unique events or phenomena that have already occurred, which cannot be controlled in experimental settings (astronomy and paleontology are other disciplines with this kind of explanations). Therefore, in these cases the aim is not to explain a pattern but a unique event or a sequence of events that took place in the past. One important component of evolutionary explanations are the antecedent conditions. These are the conditions due to which a particular outcome, and not something else took place. For example, in explaining adaptations, we should not only consider the role of natural selection, but also the antecedent conditions that made the process of natural selection possible in the first place. For instance, in a population that has no variation, there will be no selection because there is nothing to be “naturally selected.” The existence of variation in a particular environment is one of the antecedent conditions that cause natural selection, and, perhaps, adaptations. Another important component is contingency. Evolutionary outcomes are contingent per se, that is unpredictable, and contingent upon what has previously happened. Whether natural selection will take place at all, or which direction it will take is contingent in this sense, and antecedent conditions are again important here as, for instance, the outcomes depend what kind of variation was available in a population, or what kind of environment this population was living in (Kampourakis 2018).

But this is not all. There are also some less “traditional” topics that should be considered. For example, there are threshold concepts: concepts that are not specific to evolutionary theory, but that are crucial for understanding it. These include randomness, probability, spatial and temporal scales. Acquiring an understanding of these concepts is crucial for also understanding evolution (Tibell and Harms 2017; Göransson et al. 2020). To these concepts I would also add *contingency* (Kampourakis 2018) and *uncertainty* (Kampourakis and McCain 2019), and there are of course others. The important point here is that understanding evolution, and developing evolution literacy, does not only depend on learning about evolution per se, but also on understanding other fundamental concepts and features of the process. To give an example of why this matters, a common misunderstanding of evolutionary processes is that they are random and therefore one might wonder how a random process can bring about the complex structures that we observe in organisms. Understanding that evolution in general is not random, and that what is random—usually in the sense of unpredictable—are only some aspects of it (e.g., the origin of variation) is very important. Another key issue with understanding evolution is the perception of deep time; when students cannot grasp the enormous amount of time during which evolution has been taking place on Earth, they find difficult to realize how the various species and their features may have evolved.

Furthermore, there is the nature of science. It is important for students to understand that our lack of particular data does not devalue evolutionary theory; there may be fragments that we miss, but at the same time the overall picture that we have is solid. For instance, we know well from the fossil record the various hominin species that have existed in the past. That we may not know their exact relations does not change the fact that these are related in an evolutionary sense (see Wood 2017, 2019). Relevant to this is scientism and the idea that science can answer all questions. Science is a practice of methodological naturalism: it does not deny the existence of supernatural entities, but nevertheless recognizes that if one cannot study them, there is no reason to be concerned about them. This stands in contrast to metaphysical naturalism, also called philosophical or ontological naturalism, which suggests that only natural entities exist and thus denies the existence of anything supernatural. Whether a realm of the supernatural exists or not, it cannot be studied by the rational tools of science (see Chapter 7 of Kampourakis 2020a). Understanding such nature of science aspects that pertain to what science is and how it is done should be a fundamental aspect of evolution literacy and a central learning goal.

To conclude, I have argued that achieving evolution literacy requires, at the least, addressing students’ preconceptions and effectively teaching about the evidence for evolution, evolutionary concepts, evolutionary explanations, threshold concepts and nature of science. I write “at the least” because there are other elements one could add. But I believe that the ones I have mentioned here are absolutely necessary. The available research about the topics relevant to evolution literacy can be considered in order to make decision about the appropriate teaching interventions. However, this is less clear for evolutionary literacy. With this in mind, let us now see what evolutionary literacy comprises.

## Evolutionary literacy

### Apply evolution literacy to everyday life/socio-scientific issues

As I explain in the introduction, evolutionary literacy is about issues that students may encounter as citizens. Therefore, the most obvious element of evolutionary literacy is the ability to apply evolution literacy to everyday life and socio-scientific issues. In other words, it is about how to use all that I have described in the previous section (evidence for evolution, evolutionary concepts, evolutionary explanations, threshold concepts and nature of science), in order to deal with or make decisions about issues one might encounter as a citizen in their everyday life. The reason I emphasize this is that it should not be taken for granted that if one develops evolution literacy, one would also be able to apply this understanding to socio-scientific questions. Rather, additional skills are required for this purpose, and this is why developing those skills is part of evolutionary literacy. One such additional skill is about constructing arguments, which in their simplest form are claims warranted on particular grounds: a claim is an assertion that is based on particular evidence (grounds), under a specific assumption (warrant) that links the grounds to the claim (e.g., Toulmin 2003). I should note that in this case there is nothing that is really novel. In this subsection I will just describe how the components of evolutionary literacy, described in the previous section, can be applied to everyday life/socio-scientific issues. There is already a lot of discussion and research about this (e.g. Sadler 2011).

Each of the various elements of evolution literacy, described in the previous section, can be applied to particular cases, and eventually make arguments or decisions. For instance, to deal with the COVID-19 pandemic, one needs to understand some main concepts such as what viruses are, how they reproduce and how this process affects our cells, how new variants evolve and more (Rabadan 2021). It is also necessary to be able to understand concepts that are widely discussed in the media, such as “herd immunity”. In this case, it is not enough to understand what the concept means: that the more vaccinated people exist in a community, the less the virus will be transmitted from one to another person, and the more protected vulnerable people will be. It is also important to understand the feasibility and effectiveness of such a measure that might motivate reluctant people to get vaccinated (Betsch et al. 2017). Another application is on the evaluation of headlines, such as those suggesting that SARS-Co-V2 may have emerged from a biological weapons program.^1^ Whereas students cannot definitely answer this question, it is important for them to be able to evaluate whether or not this virus could have evolved by natural processes—and it was shown very early on that it indeed can, by comparing its genome to those of other well studied viruses (Andersen et al. 2020; Lu et al. 2020).

Explanations are also important for evolutionary literacy. In various countries there is discussion about antibiotic resistance. This is the phenomenon of resistant strains of microbes evolving due to the extensive use of antibiotics. Getting the explanation right here is crucial for understanding. For instance, people often think that it is the use of antibiotics that causes the emergence of the resistant strains because bacteria somehow “need” to “adapt” in order to protect themselves. However, this is wrong. What the extensive use of antibiotics causes is the prevalence of resistant strains, as these already exist, having emerged due to mutation or horizontal gene transfer. What antibiotics actually do is that by killing other strains, including those bacteria that live in our organism and naturally protect us, they provide more space and less competition for resistant strains, which in turn multiply and become prevalent.

An understanding of nature of science is also crucial. In his debate with Bill Nye “the science guy” creationist Ken Ham argued that the battle between evolution and creationism is a battle over the same evidence from the natural world. Ham argued that both evolution and creation are based on evidence from the natural world, indeed the very same evidence, and where they differ is how this evidence is interpreted in the two systems.^2^ However, this is far from accurate. Whereas this is an attempt to show that there is evidence for creation, and that it is exactly the same evidence used to support the idea of evolution, it is in fact a distortion of the habits of mind of creationists and evolutionary scientists, and furthermore of how science is done. On the one hand, creationists look for data in the natural world, which becomes evidence under their theoretical framework. But this is evidence to support a pre-reached conclusion, a version of God-has-created-the-natural-world view. Whatever evidence creationists collect is aimed at supporting this conclusion. In contrast, evolutionary scientists collect data that becomes evidence for evolution only because the latter can best explain the data. But there are disagreements in the details (the tempo and mode of evolution, among other features), and evolutionary scientists would be ready to reject their views if evidence consistently pointed to another one (Pigliucci 2002).

Related to this is the understanding of threshold concepts. Creationists often argue that complex organs such as the human eye cannot have emerged by chance. Rather, it can only have emerged through design because it is irreducible complex: if a part of a complex structure is missing, then the structure becomes non-functional. But this is not at all what evolutionary theory suggests. What is random in evolution is the origin of variation, not its outcomes. We now know that complex organs can have evolved from simpler ones, which do not even need to have performed the same function (Miller 2008; Pennock 2000). Understanding deep time is also a demanding issue for understanding and accepting evolution. Anti-evolutionists often question how it could have been possible for our species to have evolved via natural processes. The first point to understand this is to realize that if the earth had existed for 24 h, our evolution would have taken place only during the last four seconds (Kampourakis 2020a, p.134).

Applying evolution literacy to questions and issues in everyday life is an important component of evolutionary literacy. This is similar to the kinds of scientific literacy that one could envision for any science discipline. This is also what teacher education programs often address. However, I argue that what is special and distinct about evolutionary theory is that there is another component of scientific literacy, which is equally important but also more challenging for teachers to deal with.

### Draw inferences for one’s worldviews

A solid understanding of evolution should help people address issues, concerns and questions that relate to one’s worldviews. The classic case here is religion; what impact understanding and accepting evolutionary theory might have for one’s religious views. This is a topic that has been widely discussed and analyzed (for instance, see Kampourakis 2020a; Reiss, 2008). For this reason I consider another topic, which is very current and very important: racism.

The nineteenth century was an era of European dominion over the planet due to colonialism, and of the discovery of previous unexplored lands where humans lived in different way than the Europeans. The comparison of their ways of life and of their biological features to those Europeans, made many European naturalists think that there was a gradation between humans, with Europeans serving as the model, and apes, with many people from Africa and elsewhere falling in between: “Thus, in the best developed and most intellectual races, the supra-orbital ridge is smooth, well carved, and not much developed; as we descend towards the lower types, it becomes more and more marked, until, in the African and Australian heads, it has attained its maximum development.” (Nott and Gliddon 1857). This is how what is described as biological racism began: finding support in science for an unwarranted racial discrimination among human races, which would come to have a hierarchical relationship with Europeans always on the top and Africans always in the bottom.

Unfortunately, this kind of distinguishing among human groups makes sense to people even today, because of a deep psychological intuition called psychological essentialism. It consists of a set of interrelated beliefs: (i) Particular categories distinguish between fundamentally different kinds of people; (ii) The boundaries that separate these categories are strict and absolute, making the groups discrete, or non-overlapping; (iii) The individuals within the categories are physically and behaviorally homogenous; (iv) Variation within and between groups is due to internal factors (essences) that are shared within a group and which differ categorically across the groups (Rhodes and Kelsey 2020). This seems to serve, in turn, two complementary roles in: (a) the cohesion of the ingroup, that is, it serves to define who “we” are, and provides a sense of security due to one’s belonging to a group and due to the group’s uniqueness; (b) the discrimination between ingroup and outgroups, as it serves to distinguish between “us” and “others”, and justify a possible privilege status (Diesendruck 2020). Whereas many people today reject racism, psychological essentialism about social groups might make people to interpret the findings of biological research as pointing to the existence of distinct human groups.

A special case of psychological essentialism that is relevant to our discussion is genetic essentialism: the idea that genes are our deep essences. There is a lot of research on genetic essentialism, based on which it has been conceptualized as comprising four dimensions:

1. The homogeneity of genes within species or other groups, which downplays the variation among the members of the same species.
2. The fixity of genes, which entails that they are transmitted immutable, that is, less changeable and more predetermined across generations.
3. Genes as internal, single causes, which means that they directly cause observable traits; this makes them the ultimate causes, making consideration of other causal factors unnecessary.
4. The inference for the presence of gene from the observation of a related characteristic (Heine 2017; Heine et al. 2017).

In my view, this framework does not clearly distinguish between essentialism, determinism and reductionism, but rather lumps them together under the label of essentialism. Point 3 is thus very close to determinism and point 4 is very close to reductionism (see Kampourakis 2021). What is of most interest to us for the present discussion is point 1: the idea that genes form the basis for drawing the boundaries among human groups. And once such groups are found in scientific studies, they are perceived as natural.

Applying psychological essentialism to race (points i–iv above) would look like this:

i. Particular categories distinguish between fundamentally different kinds of people (Black and White people are fundamentally different).
ii. The boundaries that separate these categories are strict and absolute, so that a person who belongs to a particular category cannot belong to another (A person can be either Black or White).
iii. These categories are homogeneous, that is, their members share fundamental similarities with one another and have fundamental differences from members of other groups (White people are more similar to other White people in terms of their skin color than to Black people).
iv. All this is due to internal factors that make the members of each category what they are, which is the category essence (Black and White people have different DNA sequences related to their skin color) (Kampourakis 2023).

Both the premises and the implications of the above scheme are entirely mistaken. There are no fundamentally different kinds of people, no strict boundaries between social categories, no homogeneous social categories and no racial essences. Still, people intuitively believe that this is the case. For instance, in one study that involved 5269 self-reported African Americans, 8663 Latinos, and 148,789 European Americans living across the USA and drawn from the customer base of the test company 23andMe, most participants self-reported a single ancestry/ethnicity category. Yet, the analysis of their DNA showed a more complicated picture, and many had multiple ancestries (assuming that the respective ancestry categories exist), as shown in Table 2 (Bryc et al. 2015).

**Table 2:** Comparison of genome-wide ancestry estimates and X chromosome estimates in African Americans, Latinos, and European Americans (data from Bryc et al. 2015)

Cohort	African ancestry (%)	Native American ancestry (%)	European ancestry (%)
African Americans	73.2	0.8	24.0
Latinos	6.2	18.0	65.1
European Americans	0.19	0.18	98.6

However, people are prone to interpret research results in terms of the existence of racial essences. For instance, a widely discussed study of human DNA variation arrived at the conclusion that there exist distinct genetic clusters of humans, which differed in the frequencies of particular variable DNA markers. As the researchers noted: “Genetic clusters often corresponded closely to predefined regional or population groups or to collections of geographically and linguistically similar populations.” (Rosenberg et al. 2002). This has been interpreted by some as a confirmation for the biological reality of races. For instance, journalist Nicholas Wade wrote in his book on the topic:

Analysis of genomes from around the world establishes that there is indeed a biological reality to race, despite the official statements to the contrary of leading social science organizations. … an illustration of the point is the fact that with mixed-race populations, such as African Americans, geneticists can now track along an individual’s genome and assign each segment to an African or European ancestor, an exercise that would be impossible if race did not have some basis in biological reality (Wade 2014).

Wade’s view has received a lot of criticism (Fuentes 2014; Marks 2014; DeSalle and Tattersall 2018; Kampourakis 2023), and has made the researchers of the study under consideration to state in a subsequent article that: “Our evidence for clustering should not be taken as evidence of our support of any particular concept of “biological race”.” (Rosenberg et al. 2005). The most important issue, however, is that the study of human genetic diversity does not indicate that there exist distinct human groups. Indeed, many studies have shown that most of human DNA variation is found within human populations, rather than between them. This means that if we compared any two humans from any two human populations, we would find them to be more similar than different and we would not be able to assign them to groups if we did not see them (Lewontin 1972; Barbujani et al. 1997; Jorde et al. 2000; Rosenberg et al. 2002).

But why should biology teachers worry about these issues? Because empirical research has shown that teaching students about human DNA variation and can reduce the prevalence of essentialist views about races (Donovan et al. 2021). Aren’t racist views something that evolutionary literacy should address? The big question of course is: can they do it? It is easy to suggest adding more and more in teacher education programs and in teaching practice. Therefore, the answer to this question will have to wait until the time that we try to educate teachers who are able to address worldviews questions in their teaching and teach students how to rely on science in order to arrive at answers or conclusions. My aim so far is to emphasize that this is something worth doing.

## Conclusions

In this article, I have argued that if we want to teach for literacy in evolution, then we need to clearly distinguish between evolution literacy and evolutionary literacy. There is no way that students could deal with socio-scientific questions without the necessary understanding and knowledge of concepts and explanations. However, as I have argued in the present paper, there are important reasons for going beyond that. Evolution literacy is already something difficult to achieve, given students’ preconceptions and teachers’ difficulties. Nevertheless, the difficulties are well documented and suggestions for what to do are available (e.g., Branch et al. 2021).

But how should we deal with evolutionary literacy? For instance, in order to deal with the issue of biological racism that I described, biology teachers should be able to master a variety of topics: identity and belonging, race as a social construction, population genetics studies, and more. Can we educate teachers to deal with these issues? I definitely believe that we do, but how exactly is far from clear and straightforward. I hope that the present article will mark the beginning of a fruitful dialogue on this topic. Otherwise, we will continue teaching science content knowledge to students that will not be as useful as it ought to be for their lives. This, however, might require a total reconceptualization of science curricula that should become more trans- and inter-disciplinary, and less focused on content knowledge and exams. I think it is time to make science teaching in schools more interesting, more relevant to everyday life and more useful to our students as future citizens.

## Footnotes

1 https://www.washingtontimes.com/news/2020/jan/26/coronavirus-link-to-china-biowarfare-program-possi/; https://www.thesun.co.uk/news/11474185/pentagon-threat-coronavirus-bioweapon/.

2 https://www.youtube.com/watch?v=z6kgvhG3AkI.

## Declarations

### Data availability

There is no data related to this article to be made available.

## References

[CR1] Aldridge M, Dingwall R (2003). Teleology on television? Implicit models of evolution in broadcast wildlife and nature programmes. Eur J Commun.

[CR2] Andersen KG, Rambaut A, Lipkin WI, Holmes EC, Garry RF (2020). The proximal origin of SARSCoV2. Nat Med.

[CR3] Ball P. The gene delusion. June 2020 issue of the New Statesman, “A world in revolt”. 2020; 205.

[CR4] Barbujani G, Magagni A, Minch E, Cavalli-Sforza LL (1997). An apportionment of human DNA diversity. Proc Natl Acad Sci.

[CR5] Betsch C, Böhm R, Korn L, Holtmann C (2017). On the benefits of explaining herd immunity in vaccine advocacy. Nat Hum Behav.

[CR6] Branch G, Reid A, Plutzer E (2021). Teaching evolution in U.S. public middle schools: results of the first national survey. Evol Educ Outreach.

[CR7] Bryc K, Durand EY, Macpherson JM, Reich D, Mountain JL (2015). The genetic ancestry of African Americans, Latinos, and European Americans across the United States. Am J Hum Genet.

[CR8] Coyne JA (2010). Why evolution is true.

[CR9] DeSalle R, Tattersall I (2018). Troublesome science: the misuse of genetics and genomics in understanding race.

[CR10] Diesendruck G, Rhodes M (2020). Why do children essentialize social groups?. The development of social essentialism.

[CR11] Donovan BM, Weindling M, Salazar B, Duncan A, Stuhlsatz M, Keck P (2021). Genomics literacy matters: Supporting the development of genomics literacy through genetics education could reduce the prevalence of genetic essentialism. J Res Sci Teach.

[CR12] Fuentes A (2014). A troublesome inheritance: Nicholas Wade’s botched interpretation of human genetics, history, and evolution. Hum Biol.

[CR13] Glaze AL, Goldston MJ (2015). US science teaching and learning of evolution: a critical review of the literature 2000–2014. Sci Educ.

[CR14] Göransson A, Orraryd D, Fiedler D, Tibell LA (2020). Conceptual characterization of threshold concepts in student explanations of evolution by natural selection and effects of item context. CBE Life Sci Edu.

[CR15] Gregory TR (2009). Understanding natural selection: essential concepts and common misconceptions. Evol Educ Outreach.

[CR16] Grunspan DZ, Dunk RD, Barnes ME, Wiles JR, Brownell SE (2021). A comparison study of human examples vs. non-human examples in an evolution lesson leads to differential impacts on student learning experiences in an introductory biology course. Evol Educ Outreach.

[CR17] Heine SJ (2017). DNA is not destiny: the remarkable, completely misunderstood relationship between you and your genes.

[CR18] Heine SJ, Dar-Nimrod I, Cheung BY, Proulx T, Olson JM (2017). Essentially biased: why people are fatalistic about genes. Advances in experimental social psychology.

[CR19] Huxley TH (1863). Evidence as to man’s place in nature.

[CR20] Jorde LB, Watkins WS, Bamshad MJ, Dixon ME, Ricker CE, Seielstad MT, Batzer MA (2000). The distribution of human genetic diversity: a comparison of mitochondrial, autosomal, and Y-chromosome data. Am J Hum Genet.

[CR21] Kampourakis K (2018). Turning points: how critical events have driven human evolution, life and development.

[CR22] Kampourakis K (2020). Understanding evolution.

[CR23] Kampourakis K (2020). Students’ “teleological misconceptions” in evolution education: why the underlying design stance, not teleology per se, is the problem. Evol Educ Outreach.

[CR24] Kampourakis K (2021). Understanding Genes.

[CR25] Kampourakis K (2023). Ancestry re-imagined: dismantling the myth of genetic ethnicities.

[CR26] Kampourakis K, McCain K (2019). Uncertainty: how it makes science advance.

[CR27] Kampourakis K, Palaiokrassa E, Papadopoulou M, Pavlidi V, Argyropoulou M (2012). Children’s intuitive teleology: shifting the focus of evolution education research. Evolution Education and Outreach.

[CR28] Lewontin RC (1972). The apportionment of human diversity. Evol Biol.

[CR29] Lu R, Zhao X, Li J, Niu P, Yang B, Wu H, Wang W, Song H, Huang B, Zhu N, Bi Y (2020). Genomic characterisation and epidemiology of 2019 novel coronavirus: implications for virus origins and receptor binding. Lancet.

[CR30] Marks J (2014). Review of a Troublesome inheritance by Nicholas Wade. Hum Biol.

[CR31] Miller KR (2008). Only a theory: evolution and the battle for America’s soul.

[CR32] Norris SP, Phillips LM (2003). How literacy in its fundamental sense is central to scientific literacy. Sci Educ.

[CR33] Nott JC, Gliddon GR (1857). Indigenous races of the earth; or, new chapters of ethnological inquiry; including monographs of special departments of philology iconography, cranioscopy, palaeontology, pathology, archaeology, comparative geography and natural history.

[CR34] Oxenham J (2008). Effective literacy programmes: options for policy-makers.

[CR35] Pennock RT (2000). Tower of babel: the evidence against the new creationism.

[CR36] Pigliucci M (2002). Denying evolution: creationism, scientism, and the nature of science.

[CR37] Rabadan R (2021). Understanding coronavirus.

[CR38] Reiss MJ (2008). Should science educators deal with the science/religion issue?. Stud Sci Educ.

[CR39] Reiss MJ (2019). Evolution education: treating evolution as a sensitive rather than a controversial issue. Eth Educ.

[CR40] Rhodes M, Kelsey M, Rhodes M (2020). What is social essentialism and how does it develop?. The development of social essentialism.

[CR41] Roberts DA, Abell SK, Lederman NG (2007). Scientific literacy/science literacy. Handbook of research on science education.

[CR42] Roberts DA, Bybee RW, Lederman NG, Abell SK (2014). Scientific literacy, science literacy, and science education. Handbook of research on science education.

[CR43] Rosenberg NA, Pritchard JK, Weber JL, Cann HM, Kidd KK, Zhivotovsky LA, Feldman MW (2002). Genetic structure of human populations. Science.

[CR44] Rosenberg NA, Mahajan S, Ramachandran S, Zhao C, Pritchard JK, Feldman MW (2005). Clines, clusters, and the effect of study design on the inference of human population structure. PLoS Genet.

[CR45] Sadler TD (2011). Socio-scientific issues in the classroom: teaching, learning and research.

[CR46] Tibell LA, Harms U (2017). Biological principles and threshold concepts for understanding natural selection. Sci Educ.

[CR47] Toulmin SE (2003). The uses of argument.

[CR48] Wade N (2014). A troublesome inheritance: genes, race and human history.

[CR49] Wood B (2017). Evolution: origin(s) of modern humans. Curr Biol.

[CR50] Wood B (2019). Human evolution: a very short introduction.

[CR51] Yunis JJ, Prakash O (1982). The origin of man: a chromosomal pictorial legacy. Science.

[CR52] Ziadie MA, Andrews TC (2018). Moving evolution education forward: a systematic analysis of literature to identify gaps in collective knowledge for teaching. CBE Life Sci Educ.

